#  The use of Multidimensional Data to Identify the Molecular Biomarker for Pancreatic Ductal Adenocarcinoma

**DOI:** 10.1155/2013/798054

**Published:** 2013-09-21

**Authors:** Liwei Zhuang, Yue Qi, Yun Wu, Nannan Liu, Yili Fu

**Affiliations:** ^1^State Key Laboratory of Robotics and System, Bio-X Centre, Harbin Institute of Technology, Harbin, Heilongjiang 150001, China; ^2^Department of Gastroenterology, The Fourth Affiliated Hospital of Harbin Medical University, Harbin, Heilongjiang 150001, China

## Abstract

Pancreatic ductal adenocarcinoma (PDAC) is a lethal disease, and the patient has an extremely poor overall survival with a less than 5% 5-year survival rate. Development of potential biomarkers provides a critical foundation for the diagnosis of PDAC. In this project, we have adopted an integrative approach to simultaneously identify biomarker and generate testable hypothesis from multidimensional omics data. We first examine genes for which expression levels are correlated with survival data. The gene list was screened with TF regulation, predicted miRNA targets information, and KEGG pathways. We identified that 273 candidate genes are correlated with patient survival data. 12 TF regulation gene sets, 11 miRNAs targets gene sets, and 15 KEGG pathways are enriched with these survival genes. Notably, CEBPA/miRNA32/PER2 signaling to the clock rhythm qualifies this pathway as a suitable target for therapeutic intervention in PDAC. PER2 expression was highly associated with survival data, thus representing a novel biomarker for earlier detection of PDAC.

## 1. Introduction

Pancreatic ductal adenocarcinoma (PDAC) is a leading cause of cancer-related deaths both in China and the United States [1, 2]. The patients with advanced stage PDAC have a median survival of less than 1 year [2]. Surgery followed by cytotoxic chemotherapy or radiation is the standard treatment for PDAC. Unfortunately, this adjuvant therapy has only a modest impact on survival time [3, 4]. This situation highlights the importance of developing diagnostic biomarker for earlier detection of PDAC. 

In recent years, high throughput technologies, such as expression profiling, have provided new insights for biomarker development of pancreatic cancer [5–7]. These investigations have shown that pancreatic cancer is fundamentally a heterogenetic disease, and multiple molecular mechanisms, including the tumor microenvironment, cell adhesion-mediated drug resistance, and pancreatic cancer stem cells, contribute to PDAC progression. Although such molecular profiling analyses have produced several potential biomarkers, most of which are the lack of adequate functional significance with PDAC. Thus how those findings could be applied in daily clinical practice remains unknown. Furthermore, the remarkable genomic heterogeneity of PDAC and the small number of patients studied have hindered the advances in our understanding of PDAC.

In this study, we have proposed an integrative approach to mining novel biomarker from multidimensional omics data. This approach effectively combines patients expression profiling data with known transcriptional factor binding data, miRNAs targeting data, and KEGG pathway knowledge. This approach can produce novel biomarkers together with testable hypothesis on molecular mechanism. We have analyzed over one hundred of PDAC expression profiling arrays and large collections of TF, miRNA, and KEGG pathway gene sets. We validated several previously implicated genes with clinical significance based on literature survey and also proposed a novel biomarker for further study.

## 2. Materials and Methods

### 2.1. Gene Datasets

The gene expression data and the corresponding clinical data (GSE21501) were obtained from NCBI Gene Expression Omnibus (GEO) database, available at http://www.ncbi.nlm.nih.gov/geo/. This dataset comprises molecular profiling from 132 PDAC cancer patients in Agilent-014850 Whole Human Genome Microarray 4x44K G4112F microarray platform. Thirty samples were not analyzed in this study since the clinical data are not provided. The raw signals were normalized by quintile normalization to produce expression values.

### 2.2. Survival Analysis

Univariate Cox proportional hazards model was used to correlate gene expression data with survival data (censor status and survival days). This computation was done on all genes to genome-wide select candidate survival related genes (at *P* < 0.0001 level). Kaplan-Meier survival product-limit method and log-rank test were used to assess the differences between the survival curves of the good and poor survival patients. 

### 2.3. Gene Sets Enrichment Analysis

Gene sets can be classified into the following 3 categories (1) Genes regulated by one transcriptional factor (TF). All genes in each gene set have been experimentally verified as targets of the same TF. This curation information is collected from TRANSFAC database [8]. One hundred and six experimentally verified gene sets were used in this study. (2) Genes are predicted to be targeted by miRNA. There are many programs used to predicts miRNA targets. Previous study indicate that the intersection of PicTar and TargetScanS prediction could achieve both high sensitivity and specificity compared to other programs [9]. The update intersection of PicTar and TargetScanS predictions was used in this study. We totally obtained 715 miRNA targets gene sets. (3) Genes are included in KEGG pathway. We use the compiled human KEGG pathway, which is a collection of hundreds of manually curated pathway maps classified into subsections such as metabolism, drug development, and human disease [10]. The gene sets data can be found in Supplementary Table 1 available online at http://dx.doi.org/10.1155/2013/798054.

Enrichment analysis of gene list is a statistical technique used to reveal higher levels of functional modular changes and elucidating underlying functional mechanisms. In this study, we used enrichment analysis to correlate the TF, miRNAs and KEGG pathway with survival genes. All survival correlated genes were matched to their corresponding TF, miRNAs, and KEGG pathways. The probability for the survival gene in every gene sets was calculated using hyper-geometric function as follows:

(1) $$\begin{array}{c} p=1-\overset{x-1}{\underset{i=0}{\sum }}\frac{\left(\begin{array}{c} K\\ i \end{array}\right)\left(\begin{array}{c} M-K\\ N-i \end{array}\right)}{\left(\begin{array}{c} M\\ N \end{array}\right)}. \end{array}$$

  *x*_*i*_ is the number of altered genes in a given patient in gene sets *i*, *K*_*j*_ the number of altered genes in patient *j*,  *M* the total number of genes tested, and *N*_*i*_ the total number of genes in gene sets *i*.

The result is the probability of extracting up to *x* of possible *K* genes in *N* drawings. *P* value for every gene sets was then calculated using Fisher's test. Multiple statistical tests were controlled by false discovery rate (FDR). All of the above computations were conducted in R statistical package (http://www.r-project.org/).

## 3. Results 

**Figure 1:**
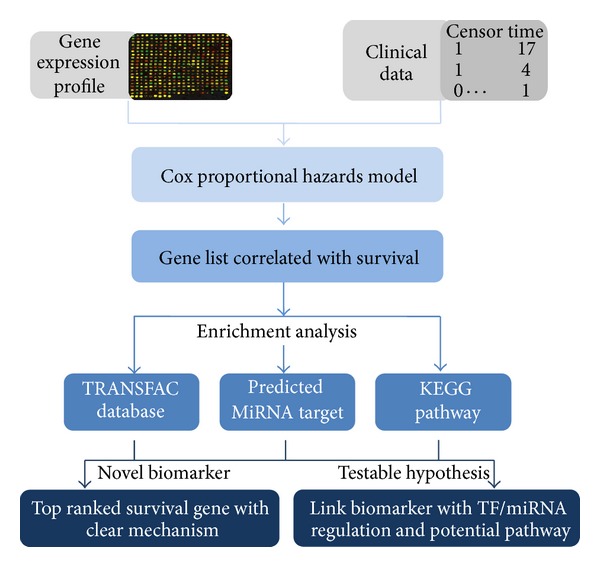
Framework of the analysis.

 The overall strategy of our approach is outlined in Figure 1. To identify survival biomarker in PDAC and its potential mechanism related with cancer progression, we initially extracted survival correlated gene by Cox proportional hazards model from microarray dataset. This gene list was simultaneously examined with TF regulation, predicted miRNA targets information, and KEGG pathways. Finally, based on the above evidence, the top ranked survival gene with clear molecular mechanism was identified as novel biomarker. Applying enrichment analysis on large-scale annotations data enables us to link potential biomarker with significantly altered TFs, miRNAs, and signal pathways. Notably, testable hypothesis can also be generated simultaneously, which greatly facilitate further functional experiments. This approach can refine survival genes with biologic significance and identify core TF, predicted miRNA, and KEGG pathways, which regulated PDAC progression.

### 3.1. Gene Expression Microarray Analysis to Identify Genes Correlated with Survival Data

We compared gene expression profile of 102 pairs of PDAC samples assayed in Agilent-014850 Whole Human Genome Microarray 4x44K G4112F microarray platform. Using univariate Cox proportional hazards model, 273 genes were found significantly correlated with patients' survival data (at *P* < 0.0001 level). These survival correlated genes (hereafter referred to as survival genes) were list in Supplementary Table 2. Inspecting this list, we found several genes, such as thymidine phosphorylase (TYMP), apoptosis-related cysteine peptidase (CASP10), and notch 4 (NOTCH4), have been implicated in cancer cells, but most of the gene are novel ones [11–13].

### 3.2. Multidimensional Analysis of Survival Genes Reveals Core TFs, miRNAs, and Pathways Contribute to PDAC

#### 3.2.1. TF Regulation Analysis of Survival Genes

In order to identify the transcriptional programs governing the expression of these survival genes, we extract the experimentally verified targets information of TFs from TRANSFAC database, and to test if the survival gene are enriched with targets genes of one specific TF. With a cutoff of FDR < 0.05, we identified 12 TFs regulation gene sets are enriched with survival gene (Supplementary Table 3). The top 5 significant TFs are summarized in Table 1. TP53 and E2F1, two well-characterized master TFs in pancreatic cancer, were successfully identified. In pancreatic cancer, these two TFs have been found to play a crucial role in diverse cellular stresses such as driving metastasis, overcoming senescence, and cell cycle arresting [14–17]. We also found three TFs, that is, signal transducer and activator of transcription 6 (STAT6), CCAAT/enhancer binding protein, and alpha (CEBPA) and progesterone receptor (PGR), are related with PDAC survival data. The molecular mechanism and functional significance of these TFs remain elusive in pancreatic cancer cells. 

#### 3.2.2. miRNAs Target Analysis of Survival Genes

The list of 273 genes was then subjected to miRNAs target enrichment analysis. Eleven miRNAs gene sets were found to be enriched with survival genes (Supplementary Table 3). Top five miRNAs involved in PDAC survival are hsa-miR-543, hsa-miR-576, hsa-miR-32, hsa-miR-545, and hsa-miR-608 (Table 1). None of them have been reported to be involved in pancreatic cancers. But hsa-miR-576 and hsa-miR-608 have been implicated in digestive system cancers. For example, hsa-miR-576 was found over-expressed in brain metastases of colorectal cancers [18]. In another report, one SNP, rs4919510 in pre-miR-608, was also associated with altered recurrence-free survival in Chinese colorectal cancer patients [19].

#### 3.2.3. Pathway-Based Analysis of Survival Genes

Discovering biologically meaningful gene patterns is highly important in analyzing genome-wide transcription profiles. Totally, we found 15 KEGG pathways enriched for survival-correlated genes (Supplementary Table 3). Many of the identified pathways in our analysis have already been implicated in pancreatic cancer. Beside, we identified circadian rhythms as a potential survival related pathway, which is consistent with recent findings that circadian transcriptional rhythms are necessary for metabolic homeostasis [20, 21].

### 3.3. Link Potential Biomarker with TF Regulation, miRNAs Targeting Information, and KEGG Pathway

**Figure 2:**
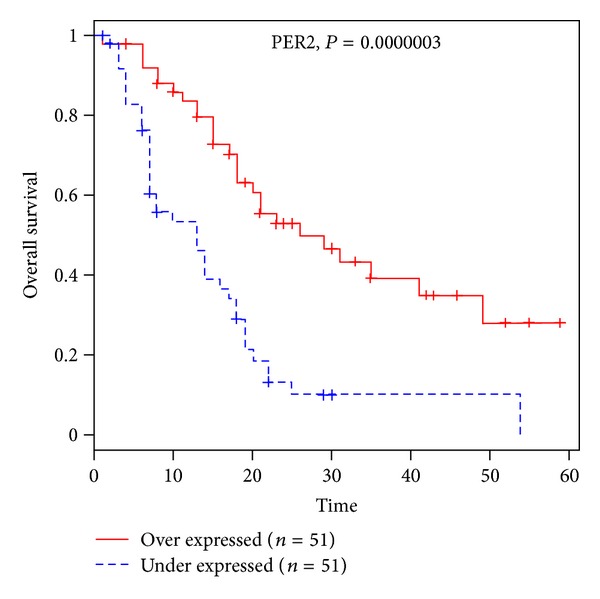
Kaplan-Meier overall survival of PDAC patients classified by high and low PER2 gene expression.

Kaplan-Meier overall survival analysis of PDAC patients expression profile revealed that PER2 was the most significantly survival gene (Figure 2. *P* < 0.0000003). PER2 gene is a key player in controlling the circadian rhythm and plays an essential role in tumor suppression. In our survival analysis, low expression of PER2 is clearly correlated with poor survival time. This is consistent with the report that overexpression of PER2 in human pancreatic cancer cells lines reduced cellular proliferation, inhibited cell-cycle progression, and induced apoptotic cell death and arrest [22]. 

**Figure 3:**
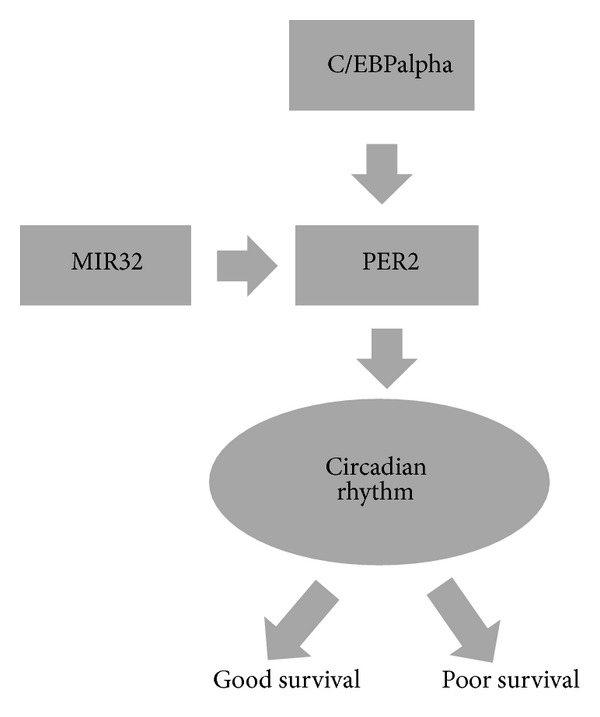
Link potential biomarker with TF regulation, miRNA targeting, and KEGG pathway. The diagram depicts putative interactions of PER2 with CEBPA, miR32 and Circadian rhythm pathway.

Based on our enrichment analyses, we found that PER2 is regulated by transcriptional factor CEBPA and predicted to be a target of miR-32. Interestingly, CEBPA, miR-32, and circadian rhythm signaling pathways are all top ranked (Table 1). Thus candidate biomarker PER2 may be regulated by CEBPA at transcriptional level and fine-tuned by miR-32 at posttranscriptional level (Figure 3.). CEBPA is a bZIP transcription factor which can bind as a homodimer or form heterodimers with the related proteins CEBP-beta and CEBP-gamma. Previous study has shown that CEBPA can bind to the promoter of leptin that plays an important role in body weight homeostasis [23]. Also, the encoded protein can interact with CDK2 and CDK4, thereby inhibiting these kinases and causing growth arrest in cultured cells [24]. In pancreatic cancer cells, Kumagai et al. found epigenetic silencing, as well as, inappropriate cytoplasmic localization of CEBPA disrupt its biological function [25]. Recently, Thoennissen et al. demonstrated direct regulation of PER2 by CEBPA in diffuse large B-cell lymphoma (DLBCL) [26]. Currently there is no report on the direct regulation of PER2 by miR-32, but miR-32 can target phosphatase and tensin homologue (PTEN) and promote growth, migration, and invasion in colorectal carcinoma cells [27]. The regulation by CEBPA and miR-32 will converge on circadian rhythms signaling pathway (Figure 3.). Mammalian circadian rhythms are an array of autonomous and autoregulatory transcriptional architecture [21]. The basic helix-loop-helix-PER-ARNT-SIM (PAS) transcriptional activators BMAL1, CLOCK, and NPAS2 activate the Period (PER1 and PER2) and cryptochrome (CRY1 and CRY2) genes, forming the core components. The deregulation of metabolic process is key event during multistage carcinogenesis. Circadian disruption accelerates cancer progression; possible due to circadian transcriptional rhythms are necessary for metabolic homeostasis. Thus, targeting circadian clocks represents a novel potential challenge for cancer therapeutics [22].

## 4. Discussion

PDAC accounts over 90% of pancreatic cancer and is a lethal malignancy with very high mortality rates. However better outcomes have been observed for smaller tumors detected at an earlier stage. For example, according to a recent analysis, the 5-year survival rates were significantly inversely correlated with tumor size [28]. This clearly indicates that detecting the PDAC at earlier stage can alter the fate of PC patients. Recently the large scale omics data present both significant challenges and opportunities for improving our understanding and treatment of this highly aggressive and lethal disease. We have adopted an integrative approach to prioritize genes of potential importance in PDAC. 

Our survival-based approach involved multidimensional analysis of gene expression, transcriptional regulation level, and miRNA level mechanisms. This novel strategy allows us successfully to discovere several known cellular mechanisms related to PDAC progression. For example, a literature survey of the top 5 pathways significantly enriched with survival genes indicated that all of them have been associated with PDAC (Table 1). It is well known that mutations in KRAS oncogene accumulate early in the disease progression and occur in almost all of pancreatic ductal adenocarcinoma (PDAC). A key downstream target of the Ras family is phosphoinositide 3-kinase (PI3K), the enzyme responsible for the generation of 3-phosphorylated phosphoinositides and the activation of Akt (protein kinase B/Akt). The PI3K/Akt pathway is responsive for the stimulation of cell proliferation and inhibition of apoptosis. Abnormal regulation of this pathway was found in at least 50% of all cancer types [29, 30]. Hedgehog signaling pathway is normally involved in patterning processes in the developing embryo, but this pathway is frequent deregulation and correlated with the mutation of the KRAS in pancreatic ductal adenocarcinoma (PDAC) [31, 32]. Glycolysis/gluconeogenesis and insulin signaling pathways, two signaling pathways related with the metabolic change, are also significantly associated with survival genes. Recent investigations suggested that strengthened glycolysis under hypoxia metabolic adaptive processes favors hypoxic and normoxic cancer cell survival and correlates with pancreatic ductal adenocarcinoma aggressiveness [33]. Furthermore, oncogenic KRAS mutation promotes metabolic reprogramming in native tumors, indicating that glucose metabolism can be exploited for therapeutic benefit in PDAC [34]. A possible association between insulin use with cancer risk has long been speculated. Recently, multiple epidemiological studies and meta-analysis revealed that pancreatic cancer risk was increased among new users of insulin or insulin glargine. Increased risk is also observed with established diabetes or new-onset diabetes [35, 36].

More importantly, our approach can link potential biomarker with TF regulation, miRNAs targeting information, and pathway mechanism into one testable hypothesis, which would not have been done based on one dataset. Specifically, we show that CEBPA/miRNA32/PER2 signaling to the clock rhythm qualifies this pathway as a suitable target for therapeutic intervention in PDAC. Our approach offers a paradigm for future larger and more complex multidimensional studies seeking to link clinical phenotype with the highly diverse molecular alterations that define PDAC or other cancer types. Although our study is a preliminary analysis of PDAC and need further verification, it provides a new avenue to find additional molecular diagnostic and prognostic markers in PDAC. 

## 5. Conclusion

PER2 may represent putative clinical biomarkers and possible targets of individualized therapy in PDAC. These results provided new insights for understanding the potential mechanisms that govern the PDAC progression.

## Supplementary Material

Supplementary Table 1 contains gene sets used in this study. It includes 106 transcription target gene sets, 715 miRNA target gene sets and 179 KEGG pathways. Supplementary Table 2 contains 273 survival gene list selected by univariate cox proportion hazards model. Supplementary Table 3 contains results from enrichment analysis of TF regulation, miRNA target and KEGG pathway.

## Figures and Tables

**Table 1:** Significant TF, miRNA, and KEGG pathway that are enriched with survival genes.

Transcription factor gene sets	FDR	References
TP53	0.0141	[14, 15]
CEBPA	0.0187	Novel
STAT6	0.0194	Novel
PGR	0.0225	Novel
E2F1	0.0329	[16, 17]
MicroRNA gene sets		
hsa-miR-543	0.0001	Novel
hsa-miR-576	0.0004	[18]
hsa-miR-32	0.0008	Novel
hsa-miR-545	0.0021	Novel
hsa-miR-608	0.0072	[19]
KEGG pathways		
Glycolysis/gluconeogenesis	0.0001	[33, 34]
Circadian rhythm	0.0001	[20, 21]
Phosphatidylinositol signaling system	0.0001	[29, 30]
Hedgehog signaling pathway	0.0007	[31, 32]
Insulin signaling pathway	0.0007	[35, 36]
